# The NAS Perchlorate Review: Second-Guessing the Experts

**DOI:** 10.1289/ehp.113-1310954

**Published:** 2005-11

**Authors:** John P. Gibbs, Arnold Engel, Steven H. Lamm

**Affiliations:** Kerr-McGee Shared Services LLC, Oklahoma City, Oklahoma, E-mail: jpgibbs@kmg.com; Consultants in Epidemiology & Occupational Health, LLC, Washington, DC

The Committee to Assess the Health Implications of Perchlorate Ingestion [National Academy of Sciences (NAS)] released its final report [National Research Council (NRC) 2005] in January 2005, recommending a reference dose (RfD) for perchlorate of 0.0007 mg/kg-day. In a commentary published online on 25 May 2005, Ginsberg and Rice (2005) criticized the adequacy of the NAS committee’s scientific deliberations, mischaracterizing the studies reviewed by the committee and second-guessing its conclusions. Ginsberg and Rice (2005) implied that the U.S. Environmental Protections Agency’s (EPA’s) previous draft RfD of 0.00003 mg/kg-day (U. S. EPA 2002)—and by inference the Massachusetts perchlorate risk assessment [Massachusetts Department of Environmental Protection (Mass DEP) 2004] that mirrored the U.S. EPA’s approach and which Ginsberg and Rice peer reviewed—is more scientifically defensible.

The NAS committee was composed of 15 leading physicians and scientists with combined range of expertise to evaluate every scientific aspect of the perchlorate database and of the U.S. EPA’s assessment of that database. The makeup of this committee and its credentials are available on the NAS website (NAS 2004). The NAS committee studied and deliberated for more than 15 months before issuing its report. Those deliberations included three public meetings during which it accepted verbal and/or written comments from the U.S. EPA, other government agencies, industry, states, environmental groups, and attorneys. After careful study and consideration of the scientific studies that formed the basis for the U.S. EPA’s 2002 draft RfD as well as the 2004 Massachusetts risk assessment (Mass DEP 2004), the NAS committee considered

several of the animal studies … to be flawed in their design and execution. Conclusions based on those studies, particularly the neurodevelopmental studies, were not supported by the results of the studies.

Although Ginsberg and Rice (2005) implied that the NAS committee should have considered the threshold for measurable iodine uptake inhibition “adverse” and that the NAS inadvertently left out the “A” in NOAEL (no observed adverse effect level), the committee decisively stated that “inhibition of iodide uptake by the thyroid clearly is not an adverse effect.” The committee carefully considered the issue of a NOEL (no observed effect level) and a NOAEL. Based on a clinical study of patients receiving perchlorate long term, the NAS established the NOAEL as 0.4 mg/kg-day (57 times higher than its identified NOEL).

Ginsberg and Rice (2005) further expressed concerns regarding perchlorate in breast milk and the subsequent possibility of decreased breast milk iodine, citing Kirk et al. (2005) and Gibbs (2004). Kirk et al. (2005) reported perchlorate and iodide levels in breast milk samples and noted that “if we take all the available data, there is no meaningful correlation between the perchlorate and iodide levels in breast milk.” The study from Chile that Ginsberg and Rice refer to as Gibbs (2004) is now published as Tellez et al. (2005). The study found that iodine nutrition of pregnant women in Chile is very similar to that in the United States. Tellez et al. (2005) found no maternal or neonatal perchlorate-related thyroid effects or decreases in breast milk iodine with perchlorate doses spanning the 0.0007–0.007 mg/kg-day range.

Ginsberg and Rice (2005) argued that perchlorate database deficiencies require an additional uncertainty factor of 3–10 because of key data gaps, citing breast milk concerns and the extrapolation from a 14-day exposure study to chronic exposure. The NAS committee (NRC 2005) considered this and concluded that

if inhibition of iodide uptake by the thyroid is duration-dependent, the effect should decrease rather than increase with time, because compensation would increase the activity of the sodium-iodide symporter and therefore increase iodide transport into the thyroid.

Evidence has subsequently shown this to be the case (Braverman et al. 2005).

The California EPA perchlorate risk assessment (California EPA 2004) relied on the same studies as the NRC report (NRC 2005). The “point of departure” was based on iodine uptake inhibition by Greer et al. (2002), and a total uncertainty factor of 10 was applied to account for interindividual variability. After reviewing the NRC report (NRC 2005), the California EPA elected not to change its risk assessment or public health goal (California EPA 2005).

In summary, the concerns presented by Ginsberg and Rice (2005) have already been addressed thoroughly by experts on perchlorate and thyroid toxicology and were found to be unsubstantiated. The NAS committee and other experts came to this conclusion based on a comprehensive review of the science in the field, not based entirely on an individual study, which has been mischaracterized by Ginsberg and Rice.
